# The evaluation of a decision support system integrating assistive technology for people with dementia at home

**DOI:** 10.3389/frdem.2024.1400624

**Published:** 2024-07-18

**Authors:** Henk Herman Nap, Nathalie E. Stolwijk, Sima Ipakchian Askari, Dirk R. M. Lukkien, Bob M. Hofstede, Nicole Morresi, Sara Casaccia, Giulio Amabili, Roberta Bevilacqua, Arianna Margaritini, Federico Barbarossa, Chien-Ju Lin, Hsiao-Feng Chieh, Fong-Chin Su, Gian Marco Revel, Ephrem Tesfay, Dorothy Bai, Claire Wirtjes, Yeh-Liang Hsu

**Affiliations:** ^1^Vilans Centre of Expertise for Long-Term Care, Digital Care, Utrecht, Netherlands; ^2^Copernicus Institute of Sustainable Development, Utrecht University, Utrecht, Netherlands; ^3^Department of Industrial Engineering and Mathematical Sciences, Università Politecnica delle Marche, Ancona, Italy; ^4^Scientific Direction, Istituto Nazionale Ricovero e Cura per Anziani (IRCCS INRCA), Ancona, Italy; ^5^Medical Device Innovation Center, National Cheng Kung University, Tainan, Taiwan; ^6^Department of Biomedical Engineering, National Cheng Kung University, Tainan, Taiwan; ^7^School of Gerontology and Long-Term Care, College of Nursing, Taipei Medical University, Taipei, Taiwan; ^8^Department of Mechanical Engineering, Yuan Ze University, Taoyuan, Taiwan

**Keywords:** assistive technology, gerontechnologies, home support, decision support system, AI, people with dementia, formal carers, informal carers people with dementia

## Abstract

**Introduction:**

With a decreasing workforce of carers and a transition from care homes to home care, people with dementia (PwD) increasingly rely on informal caregivers (ICs) and assistive technologies (ATs). There is growing evidence that ATs in the home environment can reduce workload for formal carers (FCs) and ICs, reduce care costs, and can have a positive influence on quality of life (QoL) for PwD and their caregivers. In practice, using multiple ATs still often implies using different separate point solutions and applications. However, the integral, combined use of the data generated using various applications can potentially enhance the insight into the health and wellbeing status of PwD and can provide decision support for carers. The purpose of the current study was to evaluate the use of a DSS that integrated multiple ATs into one dashboard through a small-scale field study.

**Methods:**

The current study presents the formative evaluation of a Decision Support System (DSS) connected to multiple ATs. This DSS has been developed by means of co-creation during an international project. The DSS provides an insight into the physical and cognitive status of a PwD, as well as an insight into sleep activity and general wellbeing. Semi-structured interview sessions were held in three countries (Netherlands, Italy, and Taiwan) with 41 participants to gain insight into the experiences of formal and informal carers and PwD with both the ATs and the DSS Alpha prototype dashboard.

**Results:**

The results showed that participants using the DSS were satisfied and perceived added value and a fit with certain care demands from the PwD. In general, ICs and FCs have limited insight into the status of PwD living independently at home, and in these moments, the DSS dashboard and AT bundle can provide valuable insights. Participants experienced the DSS dashboard as well-organized and easy to navigate. The accuracy of the data displayed in the dashboard is important, the context, and (perceived) privacy issues should be tackled according to all users. Furthermore, based in the insight gained during the evaluation a set of design improvements was composed which can be used to further improve the DSS for the Beta evaluation.

**Discussion and conclusion:**

The current paper evaluates a possible solution for excess AT usage and how the use of a DSS which integrated multiple AT into one single technology could support caregivers in providing care for PwD. The formative evaluation scrutinized the integration of the developed DSS and the composed bundle of ATs across diverse cultural contexts. Insights from multi-center observations shed light on user experiences, encompassing overall usability, navigational efficacy, and attitudes toward the system. FCs and ICs expressed positivity toward the DSS dashboard's design and functionalities, highlighting its utility in remote monitoring, tracking changes in the person's abilities, and managing urgent situations. There is a need for personalized solutions and the findings contribute to a nuanced understanding of DSS and AT integration, providing insights for future developments and research in the field of DSS for the care of PwD.

## 1 Introduction

In 2015, the total estimated worldwide cost of dementia care had reached $818 billion and it is expected that dementia will become a trillion-dollar disease (Prince et al., 2015). This financial burden is a significant concern, especially considering aging populations and a shrinking workforce of formal carers (FCs). In response, there has been a shift toward stimulating older persons, including PwD, to live in their own residences for as long as possible, which is also an increasing desire by many older adults themselves (Rogers and Mitzner, 2017). However, living at home for as long as possible also requires additional support for a PwD. This support can come from informal caregivers (ICs) as well as supportive technologies that can alleviate the care burden and at the same time improve the safety and quality of life (QoL) of PwD. When the transition to a care home becomes inevitable, the limited nursing workforce underscores the importance of employing technologies that can reduce the care burden.

Given the growing reliance on ICs at home, it becomes essential to examine the challenges that accompany their role. Studies have shown that supporting a PwD takes a heavy toll on ICs (van der Lee et al., 2014; Lindt et al., 2020), both in terms of economic and social costs. These costs become even higher as dementia progresses (Brodaty and Donkin, 2009). On the positive side, caring for a loved one can result in a sense of meaning, companionship, and improved QoL (Yu et al., 2018).

The long-term healthcare sector is already facing a recruitment challenge of FCs, which has further been exacerbated by the COVID-19 pandemic leading. This has led to increased labor shortages across the world (Denny-Brown et al., 2020). One contributing factor to this challenge is the demanding working conditions in the sector (Causa et al., 2021). Nevertheless, there is growing evidence that assistive technologies (ATs) in the home environment can reduce workload for FCs and ICs, reduce costs, and can have a positive influence on QoL for PwD and their carers (Madara Marasinghe, 2016; Neal et al., 2020). Examples of ATs are lifestyle monitoring systems to detect changes in life patterns (Zwierenberg et al., 2018), social robotics to support day structure (Casaccia et al., 2019; Ciuffreda et al., 2019) or senior tablets for communication and self-management (Suijkerbuijk et al., 2020). Current research and developments are predominantly focused on the design and implementation of individual ATs and their accompanying user interface (UI). In practice, however, both PwD and their (in)formal caregivers use several ATs at the same time, each with their own UI. A practical challenge that arises when multiple ATs are implemented is that different applications and technologies are needed—sometimes even only accessible via multiple different mobile operating systems—to access the collected information. This challenge underscores the practical value of integrating multiple ATs into a single and comprehensive technology, with a uniform UI, for example, a dashboard. Moreover, in the context of increasing amounts of pertinent data that may be difficult to oversee and process, FCs also seem to value the translation of data into information that supports them in their decision-making (Moreira et al., 2019). FCs may prefer using multiple ATs connected to a decision support system (DSS) to complement their clinical reasoning and strive toward a holistic view on the health status of the PwD and assess corresponding care needs (Horsky et al., 2017).

The term DSS refers to information systems. The functions of a DSS can include gathering data (i.e., sensor or manually registered data) for example, data about care needs or processes, presenting data to users (such as nurses) via, for example, a dashboard, analyzing data to generate new insights and alerts (e.g., risk calculations), selecting and providing recommendations about possible decisions and actions, and the actual implementation of decisions and actions (Parasuraman et al., 2000; Lee, 2013; Akbar et al., 2021). As Akbar et al. (2021) argue, DSSs thus far largely support the steps of analyzing data and selecting possible decisions and actions, while they could also be enabled to gather and utilize data from other sources (e.g., medical records or patient input). In long-term care, PwD and their caregivers use and interact with a variety of ATs such as tablets and monitoring systems. The use of ATs generates data about e.g., vital signs, physical activity, eating and sleeping patterns, cognitive functioning, mood, social activity, and medication intake. Feeding or integrating such data into DSSs enables these systems and their users (e.g., FCs and ICs) to utilize more varied data about a person's needs, behavior and environment to arrive at decisions about person-centered care. Moreover, these data could be utilized—whether or not in combination—by *pre-programmed, rule-based* algorithms and *data-driven, self-learning* algorithms rooted in machine learning (i.e., artificial intelligence, AI) to extract patterns and new insights from datasets that may be challenging for humans to analyze. Contemporary DSSs have already shown to support caregivers in specific aspects of the care process, such as identifying frailty, assessing dementia-related problems and suggesting suitable interventions. They may also be used for triage of health deteriorations before eventually sending a PwD to an emergency department (Iliffe et al., 2002; Lindgren and Lindgren, 2011; Kihlgren et al., 2016; Thoma-Lürken et al., 2018; Dubuc et al., 2021). Anticipated progress in AI suggests a growing role of DSSs in the proactive support of caregivers and other stakeholders in (shared) decision-making about person-centered care strategies by harnessing relevant data to provide descriptive, diagnostic, predictive and prescriptive insights (El Morr and Ali-Hassan, 2019; Mosavi and Santos, 2020).

So far, several initiatives have started with the development of DSSs and the implementation of integrated AT platforms for older people and people with disabilities, aiming for increased wellbeing, safety, independence, and confidence in their home environment. In the Vital Assistance for The Elderly (VITAL) project, a platform was developed for older people to improve personal independence and social connectivity (Hamdi et al., 2014). The project focused on using technology that could be managed by older people themselves. In the evaluation of the VITAL prototypes, users considered the applications particularly useful and reported that they could lead to improved QoL and social relationships. Another project that has aimed to support older people living alone at home is the NETCARITY project (a NETworked multisensor system for elderly people: healthCARe, safety and securITY in the home environment). The project used a monitoring system with cameras and sensors in the house to timely alert the contact persons of the elderly about abnormal activities and movements. In a study by Nap et al. (2014), the use of ATs like ReAAL was analyzed using focus group discussions with care givers, service providers as well as older adults in Spain and the Netherlands. Participants acknowledged the benefits of the technologies in improving self-management, social engagement and reducing loneliness. However, there were also some concerns regarding privacy, particularly with regard to video recordings. Usability issues were also noted, with a need for simpler interfaces. While opinions on cost varied, some participants thought the benefits outweighed the costs. In the Netherlands, the visual communication and medication reminders were particularly valued (Nap et al., 2014).

The concept of integrating several ATs in one system has been proven successful in an earlier International Active and Assisted Living (AAL) project called eWare (Casaccia et al., 2019). Combining a social robot (i.e., Tinybots Tessa) and a lifestyle monitoring system (i.e., Sensara) in the eWare project demonstrated the complementarity of different ATs and the potential of such ecosystems. While lifestyle monitoring enabled caregivers to monitor the behavior of older adults and recognize relevant patterns and unusual situations, without necessarily being at home with them (Amabili et al., 2022), the integration with the social robot enabled caregivers using the ecosystem to set more context-relevant suggestions and daily reminders by the social robot (Nap et al., 2018; Casaccia et al., 2019). Hence, integrating several ATs has the potential to strengthen the outcomes of those ATs. Furthermore, integrating multiple ATs—or at least the data resulting from their use—can be a basis for a DSS that can enable FCs and ICs to devise more person-centered care strategies. Additionally, there is a need for DSSs in dementia care as it can be burdensome for carers to use multiple technologies, applications, and UIs, and particularly in care—where workload is high—bandwidth is limited to use multiple technologies. Therefore, this paper presents the field evaluation of, a newly developed (Alpha) prototype of a DSS, which integrates data from several ATs for people living with dementia.

### 1.1 The HAAL project

To develop a new DSS, a multidisciplinary consortium was set up in the international HAAL (HeAlthy Aging eco-system for peopLe with dementia) project (Nap et al., 2022) within the AAL programme.^1^ With the HAAL project, ten organizations from the Netherlands, Italy, and Taiwan combine their expertise and experiences in the iterative development, co-design and evaluation of an AI-supported DSS for carers to gain insight into the health and wellbeing of PwD. The DSS is linked to a state-of-the-art AT bundle of products and services for PwD in various stages (see, Reisberg et al., 1982; Ipakchian Askari et al., 2024) and their (in)formal carers. The aim of the DSS is to reduce the caregivers' workload, increase the quality of care, and support independent living and QoL of PwD (Koowattanataworn et al., 2022). The developed DSS comprises a dashboard that integrates several types of data collected from PwD: their physical activity, eating and sleeping patterns, cognitive functioning, social contact, and medication intake. FCs and ICs select suitable ATs together with the people they support, based on their individual care and support needs. The ATs that can be selected are a set of AAL products and services (henceforth “HAAL technologies”), that can be employed throughout different stages of dementia and assigned according to the person's needs. HAAL technologies for example include lifestyle monitoring, daytime structure, medical dispensers, GPS trackers and serious gaming (for a complete overview, see Table 1). In addition to the integration of data from the HAAL technologies into a single dashboard, opportunities are explored to provide caregivers with only the most relevant data and to deploy AI to detect changes in patterns over time, such as changes in physical activity or medication intake. Potentially useful types of data visualization are summary overviews, alerts, predictions, and recommendations based on changes in the beforementioned data. The information provided by the dashboard can be used for early detection and prevention of health and wellbeing related issues. The targeted primary end-users of the HAAL dashboard are FCs—at the time of writing—, care professionals such as (home care) nurses, dementia care coordinators, practitioners, and alarm centralists. The use of the HAAL DSS can potentially reduce their workload. Future primary end-users of the DSS will be informal carers and likely PwD themselves.

**Table 1:** HAAL technologies available and used in the formative Alpha evaluation phase, by name, GDS scale, product group and functionalities and type of support.

**Image**	**Name HAAL technology**	**GDS scale**	**Product group**	**Functionalities and type of support**
	Tipr	2–4	Exergame	A user-centered exergaming system (palm-size, portable product) aimed to improve brain function and fine motor skills for rehabilitation. Provides precision, personal training based on strength and visual feedback to improve hand dexterity and cognitive function.
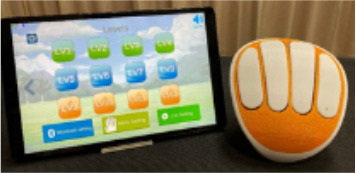				
	Compaan	2–4	Senior Tablet	A tablet with software designed for older adults. Main features are functionality management from a distance for the ICs and FCs, video calls and messaging to encourage social connection.
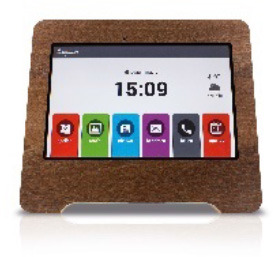				
	Medido	2–4	Medicine dispenser	An automated medication dispenser (table-top dispenser) that gives reminders for a set of prescribed medication, at the preprogrammed time. Users must confirm medication intake by pressing a button. In addition, it monitors and reports user's responses to their caregivers.
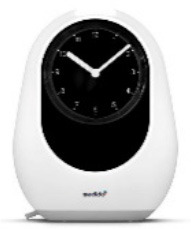				
	Kompy Pico	2–5	GPS tracker	A GPS tracker (pendant or watch). Tracks the GPS location of the PwD, ICs and FCs can build a geofence in the accompanying web application, and PwD can use an emergency button. For indoor and outdoor use.
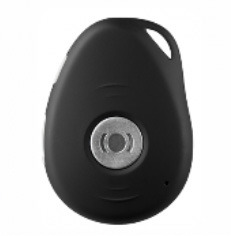				
	WhizToys	3–5	Exergame	An exergame that consists of nine portable tiles with sensors that are connected to a screen. The game combines physical activity and cognitive training, and games can be personalized. This game aims to prevent or slow down cognitive function.
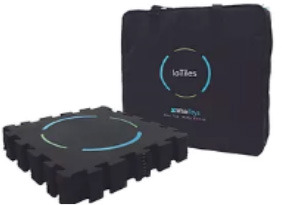				
	Tinybot Tessa	3–5	Social robot	A care robot for daytime structure, which provides verbal guidance to older adults on daily activities. The robot reads aloud written text, for example daily tasks or activities. FCs and ICs can schedule tasks and personalize spoken messages and instructions.
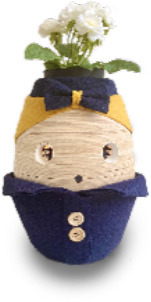				
	Sensara	3–7	Lifestyle monitoring	Movement sensors attached to walls and doors in the home of a PwD. The algorithm in the Sensara aims to detect unusual behavior. Notifications of emergencies and deviations are sent to FC of ICs.
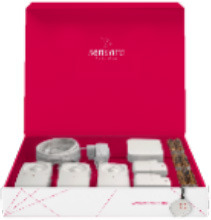				
	WhizPad	3–7	Smart mattress	A smart sensor mattress combined with transmission system. WhizPad can detect lying monitoring data on sleep, for example predict quality of sleep or help prevent bedsores.
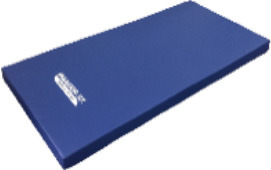				
	CogvisAI	3–7	Fall detection	A medium-size 3D smart sensor installed on a wall of a nursing home or assisted living facility. Alarms can be set for falls and other functions, for example fall prevention. Falls are recorded for further examination.
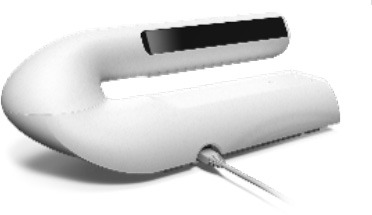				

**Figure 1:**
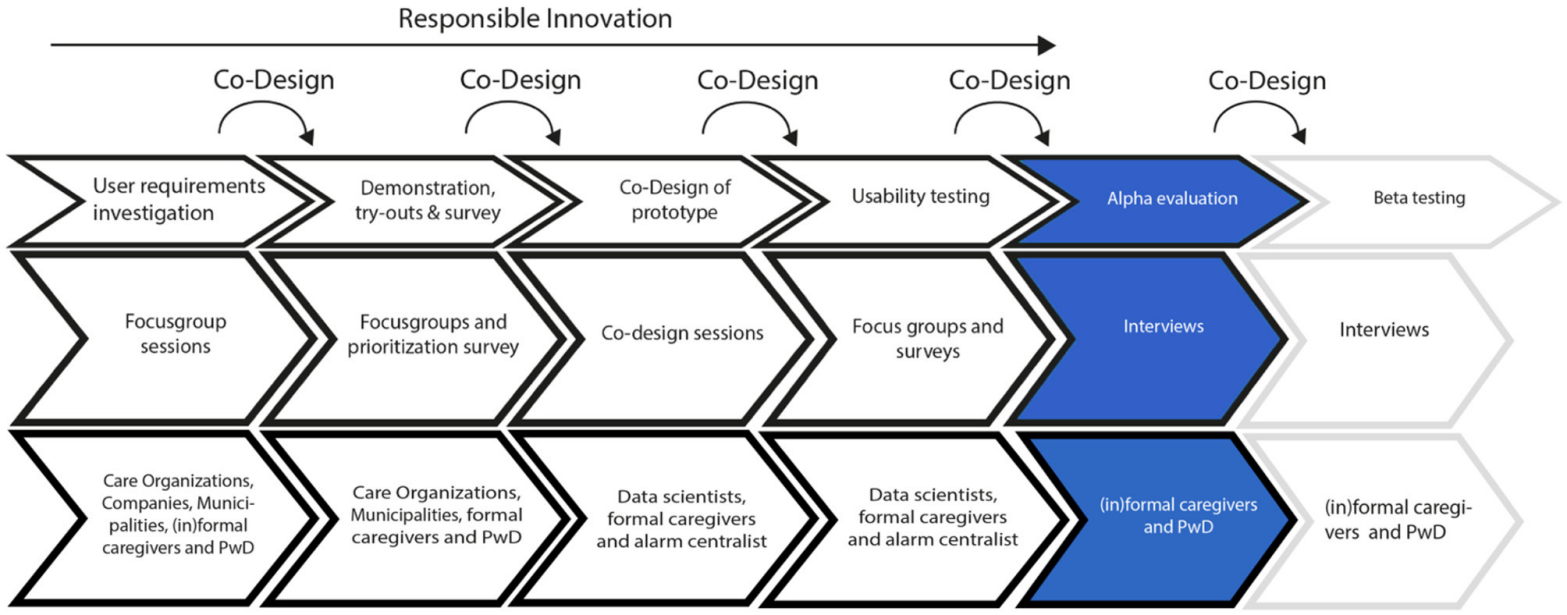
Overview of the various research activities conducted during the HAAL project.

The HAAL project prioritizes Responsible Innovation (RI), centering on ethical acceptability, societal desirability, and the sustainability of the innovation process and its outcomes, as emphasized by Owen et al. (2013) and Von Schomberg (2013). Therefore, end-users have been actively and iteratively engaged in co-design activities, playing a crucial role in identifying user needs for the DSS dashboard (see Figure 1 for an overview of the different research activities). The RI-process was led by the coordinating HAAL partner, involving multiple collective workshops and individual activities conducted among all project partners, comprising three distinctive cultural backgrounds and care systems (i.e., Northern Europe, Southern Europe, and East Asia). These initiatives aimed to increase awareness of RI and foster its integration into the research and development (R&D) of the HAAL dashboard. The reflective exercises facilitated discussions on the relevance of RI to the HAAL project, prompting considerations in the design process regarding decisions that could address societal needs and values such as privacy, autonomy, and transparency (Lukkien et al., 2023).

**Figure 2:**
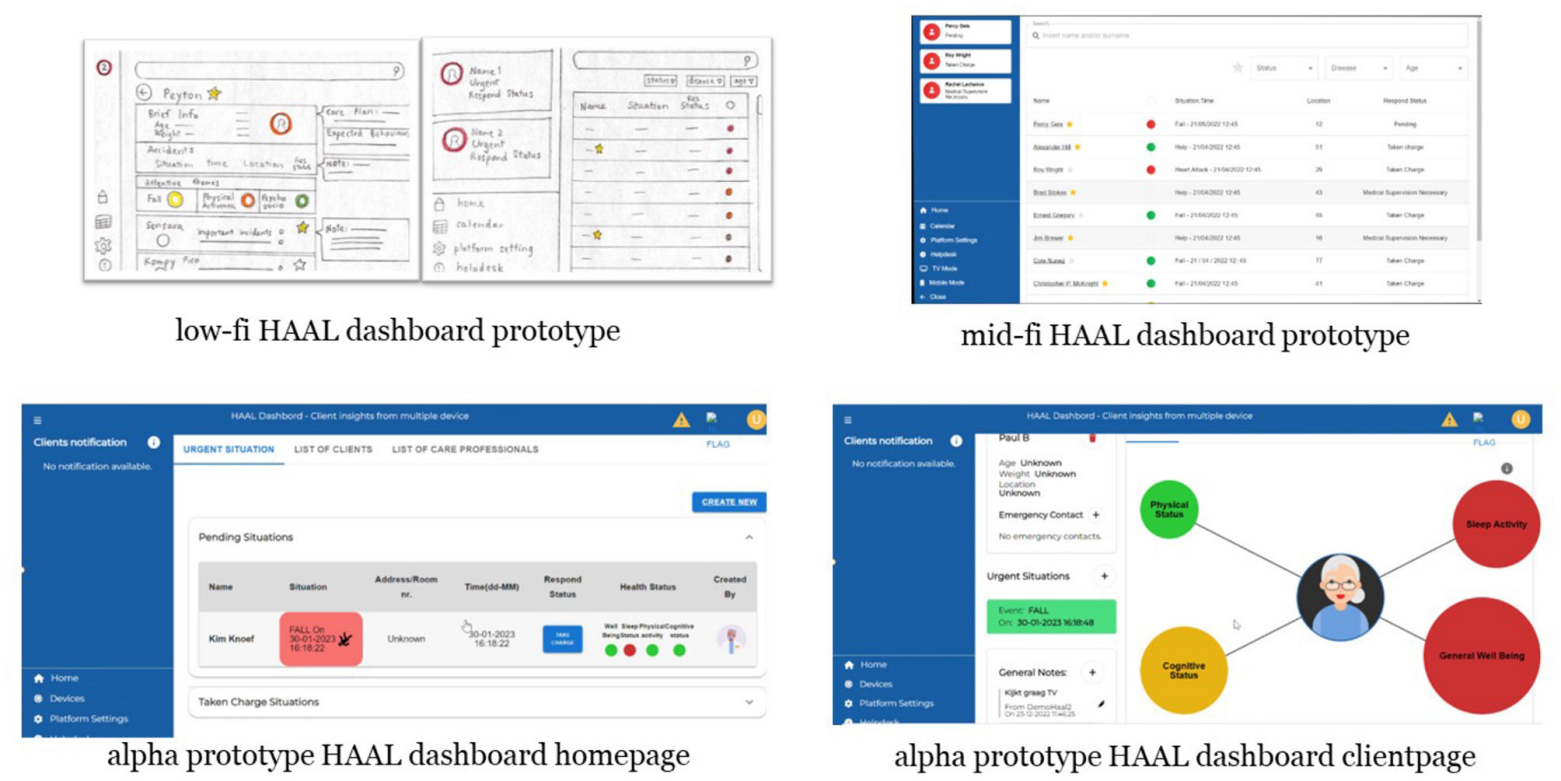
Iterative designs of the HAAL DSS dashboard, from low-fidelity **(top left)** to mid-fidelity **(top right)** to high-fidelity Alpha prototypes used in the current study **(bottom left and right)**.

The HAAL project has focused on the co-design of the HAAL DSS dashboard (see Figure 2), and this article reports on the findings of the formative evaluation (i.e., Alpha evaluation), where the first high-fidelity prototype with integrated dummy data has been used and evaluated in Dutch, Italian, and Taiwanese care organizations. The formative Alpha evaluation investigated the overall experience, usability, acceptability, and attitude over time toward the HAAL DSS system by PwD and their in(formal) caregivers. Next to the DSS dashboard, the experiences with the ATs themselves were also evaluated. The number and type of HAAL technologies selected by the participating care organization were based on their experience during meaningful try-out sessions (Cornelisse, 2024), which were conducted earlier on in the project. Additionally, based on the Global Deterioration Scale (GDS) score per technology it was listed for which need and stage it could be applicable as presented in Table 1. This information was also provided to care organizations to support them in the selection of the HAAL technologies. For more information on the HAAL project, such as deliverables and progress, see www.haal-aal.com.

### 1.2 Research aim

Based on several co-design steps, a data driven dashboard was developed. Although the prototype has been tested and evaluated during usability testing, this was in a controlled setting. Moreover, the usability testing focused solely on the evaluation of the HAAL dashboard and not on the implementation and use of the HAAL technologies. Therefore, the purpose of the current study was to evaluate the use of the HAAL platform and the HAAL technologies through a small-scale field study. The insights gained during this evaluation could then be used to further improve the HAAL dashboard for a Beta evaluation. A qualitative approach by means of interviews with (in)formal caregivers and PwD was used to answer the following research questions:

RQ1: How do formal caregivers perceive the usability of the HAAL technologies and the HAAL DSS by?
RQ2: What are formal caregivers' opinions about the accuracy and transparency of the HAAL technologies and the HAAL DSS.
RQ3: What is the perceived user satisfaction and perceived added value of the HAAL DSS for (in)formal caregivers and PwD?

To ensure that the implementation of the HAAL technologies was driven by user needs and not a technology push, PwD could, in consultation with (in)formal caregivers, decide for themselves which HAAL technologies they would like to use during their participation. In addition, the HAAL DSS was studied in a fitting setting for the care context in the three different countries.

## 2 Methods

### 2.1 Participants

In total, 41 elderly people with cognitive impairment or dementia participated in the formative Alpha evaluation. Inclusion criteria for participation were PwD aged 65 years or older who were able to give their consent for participation, had a healthy sight and hearing and were supported in the main daily activities by an informal/formal caregiver were included in the study. PwD were excluded from participation when failed to meet the inclusion criteria, or were concomitant participating to other studies. Each of the participating persons with dementia formed a triad together with one of their informal caregivers and one of their formal caregivers. Formal caregivers were nurses, district nurses, health care assistants, specialists and one case manager (in the Netherlands). The HAAL project consortium involves several care and support organizations with FCs taking care of PwD's (potential end-users of the HAAL dashboard). In the Netherlands, participants were recruited via the care organization Livio (Enschede region). In Italy, the end-users were recruited from the Neurology Unit and Alzheimer Assessment Unit (Memory Clinic) of the IRCCS INRCA. In Taiwan, Yuan Ze University (YZU) recruited participants at Bianciao Veteran Dementia Nursing Home. Via National Cheng Kung University (NCKU, Taiwan), the end-users were mainly recruited from daycare centers established by Schuhe Social Welfare Foundation. Project members in participating care organizations recruited participants using flyers, word of mouth, and demonstrations of the HAAL technologies during information markets. Before the start of the Alpha evaluation, the participating care organizations selected a subset of HAAL technologies through HAAL technology demonstrations and try-outs (Cornelisse, 2024). HAAL project members informed FC's on the functionality of the subset of selected HAAL technologies and the research process. FC's identified PwD with care and support needs that fit with the functionalities of two or more HAAL technologies. These people and their ICs were invited for the research. For information on the age and gender, Table 2 can be consulted.

**Table 2:** Gender and age of the participating PwD, ICs and FCs per country.

		**Gender (number of participants)**		**Age range in years**	**Total**
		**Male**	**Female**		
Italy	PwD	0	3	77–93	3
	IC	1	2	55–65	3
	FC	2	3	23–57	5
The Netherlands	PwD	2	3	74–89	5
	IC	1	4	unknown	5
	FC	0	5	24–48	5
Taiwan	PwD	0	5	75–91	5
	IC	3	2	37–63	5
	FC	1	4	20–42	5

One participant in the Netherlands dropped out of the study because this participant moved to a care home.

The Italian participants with dementia had a Global Deterioration Scale (GDS) ranging from five to seven, while those in Taiwan fell within the two to four range. In the Netherlands, participants were situated in the early to mid-stages of dementia as assessed by the participating FCs in the care organization. Specific GDS scores were unavailable about the Dutch participants because this organization did not apply this type of scale and did not want to adopt it for the purpose of the study. As for living arrangements, the individuals with dementia in Italy and Taiwan resided alongside their children or spouses while in the Netherlands, all participants lived independently in their own homes.

### 2.2 Materials

The HAAL bundle of ATs that was configured to the needs of the PwD was installed and implemented in a real-life setting in their homes/apartments of PwD. The HAAL technologies differed across triads. Each participant received a personalized bundle of HAAL technologies based on their specific wishes and care needs. Despite this variation, at least one complete HAAL bundle was present to supply all ATs for the triads at all three test sites. In the Netherlands, the adopted HAAL technologies comprised Kompy Pico, Sensara, WhizToys, WhizPad, Tinybot, and Compaan. Conversely, in Italy, the HAAL bundle included Kompy Pico, Sensara, CogvisAI, and WhizToys, while in Taiwan, the bundle featured WhizPad, WhizToys, Compaan, and Tipr. Additionally, a user manual was created and distributed to all participants involved in the triads. The FCs and ICs had access to the HAAL DSS Alpha prototype dashboard. The dashboard consisted of several online screens with fictional data. In addition to these technologies, the ICs and FCs were provided with a tablet from which they could navigate within the system's dashboard.

The interview questions were developed to examine the goals for the HAAL DSS during the field study. The interview protocol included questions regarding experiences using the individual HAAL technologies and the HAAL DSS. The interview topics covered potential risks and added value of using the HAAL technologies and HAAL DSS in the care process for the PwD.

### 2.3 Procedure

**Figure 3:**
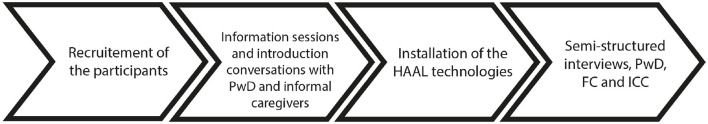
Overview of the steps taken during the study.

In this paper we report on the findings of the formative evaluations during the Alpha evaluation in 2023. The procedure for the formative Alpha evaluation was similar for all three participating countries. After inclusion and exclusion criteria were verified, participants were informed about the study and FCs and ICs signed an informed consent form. PwD were supported by their ICs and FCs to provide consent. All participants were asked to complete a socio-demographic questionnaire at T0. Information was gathered such as age, gender, living situation, use of technologies and the amount and type of care the PwD received. After the introduction phase, the treatment visit took place (baseline T1). The HAAL technologies (ATs) were installed in the homes of the PwD and the functionalities and usage were explained to them. The use of the HAAL DSS dashboard was explained to the FCs and ICs. In Taiwan, the technologies were installed in the training and rehabilitation room of the Veteran Hospital. In Italy and the Netherlands, the bundle of technologies chosen by the participants was installed in their home. In the Netherlands, follow-up measurements took place every 2 months for 6 months total, in Italy and Taiwan this was done three times in 1 month (T2, T3, T4). During the follow-up visits, semi-structured interviews were conducted with the participants, their FCs and ICs. Figure 3 shows an overview of the steps taken during the study. In addition, information was collected through the ATs' databases. In the Netherlands, some participants dropped out, because they were deceased or moved to an intramural care home. During the Alpha evaluation participants were offered the possibility to ask questions and switch in HAAL technologies. As an exit strategy, participants could keep using the HAAL technologies after the Alpha evaluation.

### 2.4 Measurements and analyses

A qualitative approach was used by means of conducting semi-structured interviews with FCs and ICs during several time intervals. Additionally, interviews were conducted with the PwD using creative methods such as conversation cards. These cards and the physical technologies served as a conversation aid to discuss their experience during the Alpha evaluation. PwD were invited to reflect on how they felt during the use of the HAAL bundle, the ease of use, trust, independence and privacy. When requested the interviews were conducted together with the IC. The interviews with FC's and IC's focused—among others—on usability, barriers, frequency of use, privacy and possible improvements of the HAAL bundle and DSS dashboard. Methods, materials and questionnaires were adapted to refer to the HAAL prototype and to match the cognitive level of the participants. Data analyses were descriptive. The interviews were analyzed by means of a thematic analysis (Braun and Clarke, 2006, 2019). A first structuring mechanism for the analysis was formed by the topic of interest: the HAAL technologies or the HAAL dashboard. As sub categories, usability and data accuracy, privacy issues and points of improvement were coded. The inductive approach of the thematic analysis also allowed for the creation of additional themes. All the interviews, across the three countries were coded iteratively by a researcher. Additionally, two fellow researchers validated the codes.

### 2.5 Ethics

The study was approved by the Ethic Committees in the individual countries and the pilot sites (Netherlands, NW2023-13; Italy, INRCA n. 3750/2023; NCKU B-ER-112-026). The principles of the Declaration of Helsinki and Good Clinical Practice guidelines were adhered to. Personal data collected during the trial was handled and stored in accordance with the General Data Protection Regulation (GDPR) 2018 (Protection, 2018).

## 3 Results

Results from the semi-structured interviews are presented per theme. The interviews were held in the native language of the participant and within the (care) context of the PwD. The quotes have been translated into English for the purpose of this article. To enhance readability, the quotes have been enriched by clarifications above the quotes and between brackets within the quotes. The information in parentheses informs about the type of participant and country, and a number is added to identify individual participants.

### 3.1 HAAL technologies

#### 3.1.1 Usability of HAAL technologies (ATs)

The section below reports on the usability experiences with the bundle of HAAL technologies (ATs) from FCs, ICs and the PwD in the three countries. We also report on the technical problems that end-users experienced with the HAAL technologies.

***Tipr (exergame)***—Both the PwD and their caregivers in Taiwan used the Tipr for finger force control training. The Tipr was found to be interesting to play with and the participants' skills improved with playing the games over 1 month of play 2-−3 times a week. However, it was unfortunate that the ICs could not perceive the improvements (yet) from the HAAL Alpha dashboard. For the Beta prototype dashboard (real-time) data needs to be integrated.

“*As clients play Tipr, we see their progress.” (Formal Carer, Taiwan, participant number 03; FC TW 03)*

***Compaan (senior tablet)—In the Netherlands and Taiwan*. **Overall, the Compaan was experienced positively by the formal caregivers. In practice they often had to answer the wellbeing questionnaire. In the Netherlands most participants did not interact with the Compaan. The caregivers responded to the messages and reminders on the tablet but they reported that it was unlikely for PwD to respond to these messages. However, there was one participant who was able to use the Compaan independently. According to the FC of the PwD, this person found the Compaan clear and easy to use, even though it was not used before:

“*When the question was asked [on the Compaan], the client pressed the button [on the screen] and answered the question” (FC NL 03)*.

One IC reported to experience the added value of the Compaan as an “additional” moment to chat with the PwD, alongside the regular visits. In Taiwan, FCs reported that the Compaan could serve well to have more (social) contact with the family of PwD.

***Kompy Pico (GPS)—In the Netherlands and Italy*. **Overall, the feedback from the ICs in Italy on the GPS device Kompy Pico was positive. Its use was perceived as supportive for safe mobility. Caregivers from the Netherlands and Italy did share that the Kompy Pico was not used to its full potential and could have been used more often by the PwD. The main common usability problem of the Kompy Pico was the size and portability of the device. One participant did not use the device because it was too large to fit in pocket pants. An FC from the Netherlands shared that it would be better to (permanently) attach the Kompy Pico to an object that the PwD always brings along with them, for example a set of keys or a belt. ICs from Italy also reported that the device might be forgotten by the PwD, and one IC stated that a device whose shape was more relatable to something familiar would be preferred.

“*I'm always afraid Mom will run away [without the GPS Kompy Pico]. The problem is that if her intention is to run away, she will definitely not take that [GPS] device, so I think the idea is good but poorly implemented”* (IC IT 02)

***WhizToys (exergame)—In Taiwan and Italy*. **The participants enjoyed playing the games and the WhizToys was used for lower extremity training. In Italy, two participants also had fun playing the games offered by the WhizToys platform. One IC was happy that the participant had something stimulating to engage with and keep her mentally and physically involved. However, some technical problems were met.

“*My mother had a lot of fun with the WhizToys, although it had several technical problems.”* (IC IT 02)

***WhizPad (smart mattress)—In Taiwan and the Netherlands*. **There was a high demand for gaining insight into the sleeping behavior during the night. However, some participants found the WhizPad mattress slippery or thought it raised the bed too much to get into bed safely. Multiple participants mentioned that the battery of the WhizPad indicated being low while there still was battery life. Also, there were some concerns whether the data was valid because other people present in the house might have generated them, for example when the bed was in the living room.

“*When the visitors are sitting on the bed [which is in the living room], it seems as if sir is sitting on the bed himself” (FC NL 02)*

***CogvisAI (fall sensor)—In Italy*. **One IC reported that the CogvisAI was one of the most useful technologies in the bundle. However, a limitation of the technology was the small coverage radius of the technology, which should be wider to ensure more safety. Also, it would be necessary to have one technology in every room of the house to maximize the utility.

“*The fall detection system [CogvisAI] was useful, however, should have a wider range [to detect falls]”* (IC IT 01)

***MEDIDO (medicine dispenser)—in Netherlands*. **Regarding the medicine dispenser Medido, FCs and ICs in the Netherlands were positive about the technology. One participant reported to be satisfied with using the Medido because it provided tangible evidence of its effectiveness, unlike the other HAAL technologies that monitored him. One IC indicated that even though the PwD was correctly reminded of the medication intake, it was difficult to validate if they actually took the medication. It could still be possible that the medication was taken out of the Medido, but placed on a table or other location and that the concerning PwD had forgotten to take this medication.

***Regarding all technologies—***A number of participants from the Netherlands conveyed instances where the charging of HAAL technologies was inadvertently overlooked, often attributable to misplacement or inadvertent disconnection of the technologies. In Italy, users occasionally encountered disruptions in data acquisition due to inadvertent disconnection of power to the internet modem.

#### 3.1.2 Difficulties and accuracy of HAAL technology data

During the user trials, we encountered several implementation challenges. First, regarding the lifestyle monitoring system, several FCs indicated the importance of knowing the position of the different sensors. FCs shared that it was sometimes challenging to find the appropriate position of the sensor as it is important to maximize the space that can be monitored by the sensors, in order to minimize errors and/or blind spots. Another challenge was related to the presence of more than one person in the home of the participant. Although all PwD lived alone, there were frequent visits from ICs or FCs. In such situations, the system could temporarily be turned off. However, in practice the system mostly remained on, which resulted in misinformation in the dashboard. It was mentioned by an IC that other FCs would also open the refrigerator door. This resulted in incorrect information regarding the frequency of the refrigerator being opened by the PwD. Concerns were expressed by users from the Netherlands and Italy that if a someone would receive many visitors or over a longer period, important data on their movements could be lost. The technology cannot distinguish the intended user from other people in the house. In addition, the presence of pets could alter the correct recording of data.

“*If the sensor detects any movement of any person, we will not be able to actually understand how much movement my mother makes in the house”* (IC IT 03)

A similar limitation was observed with the WhizPad. The WhizPad lacks the capability to discern whether one or two individuals are seated on the smart mattress, consequently leading to the dissemination of inaccurate information. Moreover, challenges surfaced during the integration of Tinybots Tessa, with a formal caregiver from the Netherlands elucidating an instance where a participant's ability to hear messages delivered by Tinybots Tessa was compromised when they were outside their room or living area.

#### 3.1.3 Overall satisfaction with the HAAL technologies

In the Netherlands, both IC6 and IC3 were positive that the HAAL technologies could support them in their care responsibilities. They explained that the HAAL DSS dashboard could provide additional insight into situations that they would normally have no information about because they would not be present in the home of the PwD. The ICs reported that the use of the HAAL technologies, such as lifestyle monitoring, could support in the early detection of a bladder infection/pneumonia or provide a notification of fall incidents. Moreover, it was mentioned by IC6 that the collected data could provide a more accurate perspective on the behavioral pattern of the PwD, which is desired when it comes to monitoring food patterns. However, it was also reported that some technologies were not easy to use. In Italy, ICs experienced some obstacles when using the WhizToys. One of the problems was caused by the frequent disconnection from the internet. Nevertheless, ICs indicated that the WhizToys had a number of interactive games that were interesting. An FC in Italy emphasized the usefulness of the GPS Kompy Pico in nursing homes that have an outdoor space where people are free to move around. Another FC stressed the usefulness of WhizToys as a physical-cognitive rehabilitation game if the activities are conducted in the presence of a supervisor.

“*In the nursing home where I work there is a garden and patients can go there freely, so a tracking system like Kompy Pico is useful” (FC IT 04)*

The participants from both the Netherlands and Italy appreciated the HAAL technologies, yet their satisfaction was lower than that of the people taking care of them. Overall, the participants seemed to prefer the HAAL technologies that had a small impact on their home environment and daily life, for example passive sensors. However, there were also some technologies that had some impact on the home environment of the participants, for example, the Compaan (senior tablet) has a shining bright light in the bedroom, and Tinybots Tessa produces sounds. The perceptions of the Kompy Pico (GPS) were mixed. On the one hand, results showed that carrying the GPS tracker brought feelings of comfort. On the other hand, it was reported that the need of the GPS tracker might be unnecessary because the participant was well enough to take their usual stroll. One participant expressed an overall neutral attitude toward the technologies and did not adjust his lifestyle to the technologies. In Italy, some of the participants were satisfied that their family members could be more relaxed about their situation, yet the technologies in the home created some concerns for two of the participants. For example, they were bothered by the (high) number of technologies scattered around the house, and they feared inadvertently breaking something.

“*There are too many wires, too many things in the house”* (PwD IT 01)

In contrast, ICs and FCs from the Netherlands assumed that most PwD would not be aware of the presence of the technologies. In Taiwan, the participants in the daycare center indeed positively and happily used Tipr. The gamified interface made them feel like they were playing games.

“*Is it my turn to play Tipr?”* (PwD TW 01)

One FC at the day care center explained that for some persons with dementia their physical condition improved by using Tipr and WhizToys.

“*Client's physical condition improved after training with Tipr and WhizToys. This will help reduce the care burden on caregivers in the daycare center.”* (FC TW 03)

### 3.2 HAAL DSS dashboard

#### 3.2.1 Usability of the HAAL dashboard

Overall, participants were positive regarding the usability of the HAAL DSS dashboard. ICs and FCs in both the Netherlands and Italy experienced the HAAL dashboard and its functionalities as clear and intuitive to use, with smooth navigation. Specifically, three FCs from the Netherlands mentioned the layout of the user page to be pleasant and intuitive.

“*Also clear, it is self-explanatory, if you work with it more often you will know where to click”* (FC NL 04)

Even though the dashboard did not (yet) display the accurate data collected by the installed technologies, most FCs in Taiwan were positive regarding the placement of the sensor data in the HAAL dashboard.

“*The dashboard is comprehensive. It [the dashboard] is very clear.”* (FC NL 06)

“*The platform is simple and easy to use. Using the HAAL platform helps me understand the client better, thus increasing my empathy for him.”* (IC TW 04)

Italian caregivers were also positive about the current user interface that was used to describe the status of the PwD. They expressed that the graphics and the “circle” format provided clarity and allowed the caregiver to have an immediate overview of the person's wellbeing, making monitoring more efficient and effective.

“*The colored circles are very useful, because they give an immediate general idea of the patients”* (FC IT 01)

Moreover, most ICs and FCs suggested that they found it logical and clear how they could navigate through the dashboard. However, one IC from the Netherlands mentioned that it was not fully clear how to interpret the red color of the “circle” regarding the sleep. Another IC pointed out that the dashboard still had some spelling and translation errors. Moreover, it was confusing that all participants were visible in the user list, as this reduced the clarity of the dashboard.

#### 3.2.2 Frequency of use of the HAAL dashboard

ICs and FCs in Italy reported that they checked the dashboard almost every day. Two Italian FCs used the dashboard for just a couple of min while three other FCs used it between 30 and 60 min. In Taiwan, the HAAL dashboard was used 2–3 times a week, whenever (rehabilitation) training sessions took place with the Tipr and WhizToys.

“*I checked the [HAAL] app about once every two days”* (IC IT 02)

“*30 min a day, at the beginning and end of my work shift”* (FC IT 03)

“*Since the technologies used in the daycare center are not yet fully integrated, I rarely check the HAAL dashboard.”* (FC TW 03)

In the Netherlands, the frequency of use of the HAAL dashboard was relatively low. Some caregivers explained that they did not use the dashboard that often because it showed fictional data. Nevertheless, some of them logged into the dashboard a couple of times and easily navigated through it. They reported that if this system was fully developed with real-time data, the dashboard would be checked on a regular basis.

#### 3.2.3 Accuracy of the HAAL dashboard data

Regarding data accuracy and user trust in the HAAL dashboard, Italian FCs affirmed the data's accuracy and expressed intent to integrate the data into their clinical practice. Conversely, several Dutch FCs noted inconsistencies in the logical sequencing of activities within the lifestyle monitoring section of the dashboard. Specifically, they highlighted instances where notifications of activities appeared illogically ordered, underscoring the need for data annotation before analysis and presentation. They proposed to implement a manual button enabling caregivers to confirm or discard specific situations, enhancing data accuracy and trustworthiness through user annotation or 'human-in-the-loop' involvement. FCs from Italy considered the dashboard as a means of *supporting* their clinical decisions, and they reported that their professional judgment was decisive.

“*Yes, they [dashboard data] are suggestions, obviously then my judgment as a professional will be more important”* (FC IT 02)

As for the Italian ICs, all agreed to consult the dashboard and follow its advise. However, they stressed that it is essential to recognize that the DSS primarily serves as a supportive tool providing a general check on the person's wellbeing.

#### 3.2.4 Overall satisfaction and perceived added value HAAL dashboard

The overall satisfaction and added value of the HAAL dashboard for Dutch and Italian FCs and ICs was high. FCs and ICs indicated to have limited knowledge on the health status of the PwD in their home context. They wanted more insights into the moments that they were not physically present in the person's house. Two FCs from the Netherlands reported an interest in the GPS data. One specifically wanted to know if the person they were taking care of went to the appointments that were added to the agenda. Another FC was especially interested in the lifestyle monitoring data on the dashboard.

“*Yes, I find it especially interesting to see how often she gets out of bed and what her night rhythm is exactly.”* (FC NL 04)

FCs and ICs noted that gaining insights could enable more appropriate actions. In one case, lifestyle monitoring data (specifically toileting) informed multidisciplinary consultations, leading to interventions such as urine sample collection. According to a Dutch FC, a more functional benefit of the DSS dashboard was that without it you would need to separately log into several webapps and applications where as the HAAL dashboard provides a direct overview of the data from different technologies.

“*If you use healthcare technology on different websites, you have to use a different login each time. A while ago I wanted to log in to Sensara but first I spent an hour looking at how to log-in to see which apps I needed. So, if you have everything on one dashboard, it is more clear and easier for ourselves”* (FC NL 04)

In respect to the added value, Italian ICs shared that the use of the dashboard could reduce both their workload and that of the professional. In addition, FCs shared that the dashboard could be useful as a comprehensive monitoring tool, allowing them to focus first on those people that need immediate support, i.e., triage of a PwD. The DSS could support in managing people and simplify the work dynamics of FCs, especially because of time savings, a greater peace of mind and an overall sense of security. The results of the research underscored the vision of the dashboard as supporting the daily monitoring by carers.

“*I could immediately get an idea of how all the clients are doing, so that I could arrange rounds of visits according to priority”* (FC IT 03)

“*I believe that the work commitment can be reduced, and that time saved can be used in other work needs”* (FC IT 04)

However, one Italian IC highlighted the need for technical robustness to ensure user satisfaction and effective use of the system.

“*I didn't find it [dashboard] very useful, the basic idea is good, but there were too many technical errors”* (IC IT 02)

In Taiwan, FCs reported that the communication with the families of the PwD improved. In the Netherlands, a FC valued the dashboard as a communication tool.

“*The factors [data and recommendations] from the dashboard could be used as a conversation guide for example with family. It could be an easy approach to start a conversation”* (FC NL 03).

Moreover, it was expected that by selecting technologies that could facilitate physical and cognitive training, the health status of the PwD could also be improved. Furthermore, this could prevent health issues and, as a result, reduce the workload of caregivers. Both the PwD and the FCs expected improvements in the hand and lower extremity after the training period of 1 month. During testing, most PwD found Tipr and WhizToys interesting to play with. One formal caregiver noted: “*It is seen that the skill of playing with Tipr and WhizToys improved noticeably in the clients; however, it is unfortunate that their family (informal caregivers) cannot perceive it from the dashboard””* (FC TW 01).

#### 3.2.5 Clarity and applicability of the HAAL dashboard functions

The main finding regarding the HAAL dashboard highlighted its applicability for gaining insights into a person's behavioral patterns. FCs and ICs from the Netherlands and Taiwan expressed a desire for systematic recording of the overall condition of the PwD, with potential benefits for caregivers. Dutch caregivers sought separate access to data collected by individual technologies. FCs and ICs mentioned the potential benefits of utilizing the HAAL technologies' data to predict and stimulate future behavior to enhance the QoL. Specifically, being able to monitor bathroom visits or wandering, was deemed beneficial for early detection of issues like bladder infections.

“*Once all technologies are integrated, I hope families will be able to see how clients are doing at the daycare center. We expect the HAAL dashboard to enhance communication between families and formal caregivers.” (FC TW 01)*

According to the FCs, caregivers primarily communicate through direct messages or phone calls, rendering the dashboard's internal messaging function potentially redundant. FCs suggested that having essential contact information, such as phone numbers displayed on the dashboard, would suffice.

#### 3.2.6 Points of improvement for future development of the HAAL DSS dashboard

While considering the points of improvement as mentioned before, there were additional improvement points reported by Dutch and Italian FCs and ICs, specifically regarding the implementation and end goals of the HAAL dashboard.

First, based on the remarks made by two FCs and one IC regarding their anticipated frequency of use and ultimate responsibility, it is important to decide if the HAAL dashboard will be used as a notification system or a consultation platform. Moreover, it is important to determine which parties and how many individuals will get access to the dashboard. Secondly, two FCs and one IC were concerned that caregivers and PwD might not have the proper knowledge on how to operate the HAAL technologies or experience difficulty interpreting the information on the dashboard. This would make it difficult to integrate the HAAL technologies and the dashboard into actual health care practice. They argued that training and support should be provided for implementation and usage.

“*If we are to work with this technology [Compaan], there must be a clear policy. So, everyone can be on the same page and knows what to do. Some guidance is needed there.”—(FC NL 03)*

Similarly, the need for training and support would also be applicable for the expected actions that need to be taken by the people who are responsible (e.g., FCs and ICs) when receiving certain alarms/messages from the HAAL dashboard.

Additionally, one FC mentioned that the dashboard should not interfere with the professional and logical knowledge of healthcare workers. On the contrary, it was explained that FCs should be able to judge circumstances according to their professional knowledge instead of blindly trusting the dashboard and its technologies.

FCs from the Netherlands reported that it would be more efficient to link the electronic health record (EHR) of the PwD to the HAAL dashboard directly. This way formal caregivers could report directly to the EHR.

Dutch, Italian and Taiwanese FCs and ICs suggested that the usefulness and usability of the HAAL dashboard could be improved by making some practical design changes to the dashboard. The design suggestions per country are presented in Table 3 below.

**Table 3:** Overview of suggested design improvements for the HAAL DSS dashboard per section, made by FCs and ICs (Netherlands, Italy and Taiwan).

	**Netherlands**	**Italy**	**Taiwan**
**General**	***Linguistics & terminology*** • Use the correct medical terminology when describing situations, conditions or other descriptions. (FC) • Allow the user to change the language of the dashboard to their preferred language and enhance spelling. (FC & IC) ***(Transmission of) information*** • Send alarm notifications through an (phone) application (IC) • Connect the EHR to the HAAL dashboard directly. This way the FCs can report directly to the EHR. (FC) • Add an explanation about why a certain message pops up on the dashboard. It is desired to know how the dashboard (i.e., algorithms) came up with certain statistics. (IC)	***(Transmission of) information*** • Add a page that lists all the technologies and their features. This supports FCs in finding the most suitable technologies for PwD. ***User interface*** • Make the user interface of the dashboard more accessible.	***(Transmission of) information*** • Create a dashboard that would be compatible with a mobile phone. For FCs in the daycare center, it will be more practical to check the status of PwD through their mobile phones. ***User interface*** • For the login process, it would be helpful if users could view or change their passwords.
**Homepage**	•Add a button to give the caregiver a manual option to discard (or confirm) a certain situation (FC)	•Improve the management of urgent situations. It was suggested to add the option to delete a resolved situation.	•Provide a function to view the instruction manual on the dashboard for users who are less tech savvy.
**PwD page**	•Add the PwD's medical condition on the dashboard to provide context of the health status (for example when a PwD has diabetes). (FC) • Allow adding notes to the PwD's overview in the dashboard, for example small personal traits. (FC, IC) • Add a section to present the data collected by each technology separately. (FC)	•Allow FCs to choose which health information is displayed for a particular patient. For example, add information such as the diagnosis. • Add a section where primary caregivers can insert which therapy a patient is following. • The profile picture should be of the PwD, instead of an avatar.	•Offer the possibility to record notable events (e.g., medical treatments) and provide notifications to caregivers.
**‘List of care professional' page**	•Remove the function of the dashboard that allows caregivers to contact each other through the HAAL platform. Having the required contact information, such as the phone number presented on the dashboard would be sufficient. (FC)	•Allow professionals to manually add additional information about the caregiver, such as phone contact, profession, days of reception. This information could be added in a separate tab. • Add a search tool, where one can search for caregivers based on their name.	

### 3.3 Privacy issues regarding the HAAL technologies and HAAL dashboard

In the Netherlands, the PwD primarily raised privacy concerns. Regarding lifestyle monitoring, some people perceived that using the system would be an infringement of their privacy. One participant expressed having the feeling of being monitored by the sensor at the door, which caused a feeling of unease. Similarly, in Italy several PwD raised concerns about being monitored in their daily activities. However, some formal carers valued having control via a dashboard above privacy.

“*No worries about' privacy, it is worth having your data in a system if it means you have more control”* (FC IT 01)

One PwD from the Netherlands did not experience any privacy issues, as the HAAL technologies did not make use of cameras for the monitoring activities.

“*You are just being monitored. I don't mind. In the beginning I found it a bit difficult that I was being watched. Now I don't feel that way anymore. Why not? The technologies are just there now, and I just leave them alone.”—(S NL 03)*.

One Dutch FC wondered whether caregivers should continuously be aware of the whereabouts of the people they take care of.

“*As a healthcare professional and case manager, it is an invasion of privacy to keep an eye on where your clients hanging out. When I'm that age, and I have a technology that tracks my movements, I'd rather have only my children seeing that. For me as a case'manager I don't think there is any added value to it.”—(FC NL 03)*.

## 4 Discussion

The formative multi-center and multi-cultural evaluation of a DSS (decision support system) and connected bundle of Assistive Technologies (ATs) for (PwD) in the home setting offered relevant insights on the stakeholders' (PwD, ICs and FCs) experiences. Insights were gained over time, encompassing the overall user experience, the navigational efficacy of the DSS dashboard, as well as considerations of acceptability and attitudes toward the DSS dashboard and ATs. In the following sections, we will first discuss the experiences with the DSS followed by the ATs. Thereafter, we will discuss limitations and provide suggestions for further research.

### 4.1 Experiences with the decision support system: usability, accuracy and transparency of data

The results showed that both FCs and ICs from all three countries were positive about the design and functionalities of the DSS dashboard. They appreciated a single dashboard for remote monitoring, the ability to show gradual changes in the PwDs' physical and cognitive abilities They also liked to receive indication alerts of urgent situations and information to predict and prevent health issues. The design of the DSS dashboard was considered usable, clear and intuitive by FCs. The organization of functions within the dashboard was perceived as logically structured, easing seamless navigation and operation. Despite positive experiences, improvements were needed for the DSS dashboard, such as precise terminology, real-time data, the provision of a comprehensive instructional manual, and clear information about responsibilities (e.g., who should follow up on an alarm). Next to these improvements, both accuracy- and confidentiality of data were principles that FCs and ICs found important in the design and use of a DSS. It was stressed that information provided by the DSS dashboard should not lead to any faulty judgements made by caregivers, and that both the data and the algorithms processing data should therefore be accurate, without biases and without being too directive. This resonates with principles for ethical and responsible AI, such as accuracy and fairness, as promoted by organizations such as the European Commission (2019) and the World Health Organization (2021). Also, this implies that some transparency should be provided about the underlying functioning and algorithms of DSS to ensure that users can properly understand how specific insights are generated. This helps them to assess the applicability and relevance of these insights in the context of individual PwDs.

Besides the need for transparency of the algorithms, the data in the DSS should be accurate in order to be used in health care decision making. Only accurate data would help to choose proper interventions for e.g., training cognition, physical activity and monitoring mobility and sleep quality. The care- and client's context should also be accounted for in the DSS to enhance decision making. Contextual information could involve PwD characteristics such as their cultural and socio-economic background, as well as caregivers' own observations or interpretations. Such information could provide a broader perspective on the relevance of DSS insights, and could be supportive for caregivers to develop a nuanced understanding of a PwD's situation, and care and support needs. In the forthcoming iteration of the DSS, enhancements will include the integration of data visualization features to ensure a more detailed insights to users. FCs from all three countries were already positive regarding the clarity and placement of the sensor data in the DSS. In order to validate the data in the DSS and increase the accuracy of the data, feedback options in the DSS need to be added e.g., functionality to annotate or rate the data via the DSS dashboard. Leveraging these feedback options, users can relay insights and observations to developers, thereby facilitating iterative improvements to the underlying algorithms. Such iterative refinements hold promise in minimizing instances of erroneous data interpretations, consequently mitigating the occurrence of false alarms or data omissions, as posited by Swets et al. (1961).

### 4.2 Experiences with the assistive technology bundle: benefits, cultural, and contextual differences

Multiple different ATs are connected to the Alpha DSS prototype providing support to PwD in both physical and cognitive domains, facilitating activities such as training, medication management, and fostering remote social interactions with FCs and ICs. In respect to the experiences with the bundle of ATs, it was found that overall, the experiences were positive. FCs and ICs felt supported by the ATs, particularly in localization of the PwD and monitoring their lifestyle, such as eating and sleeping patterns, as also found by Zwierenberg et al. (2018). Central to this support felt during the use of the HAAL AT bundle was the flexibility inherent in selecting ATs tailored to individual needs, desires, and capabilities, i.e., a technology pull rather than a technology push. Nevertheless, the introduction of certain ATs was met with reservations among the PwDs in the Netherlands and Italy, who perceived them as intrusive. According to the PwDs, the ATs had an impact on the physical context of the home environment, such as the large amount of equipment with wires, the bright light from the senior tablet and replacing a regular mattress by a smart one. Moreover, ATs can stigmatize people by age or disease (Parette and Scherer, 2004), which might also have influenced the PwDs' experience and acceptance of the ATs. For ATs in general, it is therefore advised to embrace the “Warm Technology” design process, which is sensitive to the possibilities and unique qualities of old age—personal, affective, social, contextualized, and embodied (IJsselsteijn et al., 2020). Warm Technology aims at improving (or remaining) QoL by supporting and enhancing human potential, dignity, social connectedness, and self-reliance.

As discussed before, the accuracy of data is crucial in health care decision making. The study shows that the individual AT data and the aggregated processed data by AI algorithms presented in the DSS dashboard are highly dependent on the data from the ATs in context. Misplacement of lifestyle monitoring sensors, as well as the presence of animals or multiple people in the house, can cause false alarms or misses. Similar reductions of accuracy in data were present with the smart mattress, which cannot differentiate (yet) between multiple people sitting on the bed (e.g., a formal carer and the PwD). Reduced accuracy can also be caused by users that do not perceive messages from the social robot or medicine dispenser, possibly because they are not present in the room, have hearing problems, and/or because of sound disturbance or interference. There is a delicate balance between monitoring a PwD for safety, and their need for autonomy and privacy. Privacy concerns among the PwDs underscored this delicate balance, particularly in relation to the lifestyle monitoring sensors. Therefore, we reflected on whether continuous monitoring is worth the privacy infringement. The findings show that opinions on this matter differ among various stakeholders and that it is dependent on whether one sees the application of the AT as infringement of privacy. It seems that for now it needs be decided per individual case whether the PwD would experience the AT as infringement and offer them the opportunity to decide if the technology can be installed in their home. Moreover, it is essential that an AT is only applied when there's a clear need, for example, in the case of a identified safety risk. It is noteworthy that the PwDs from Italy were satisfied with the idea that family members are supported by the DSS and Ats. We speculate that is possibly due to the remote care- and monitoring possibilities. This finding underscores the nuanced interplay between technological intrusion and the broader socio-cultural context within which these assistive technologies are integrated.

Different users valued and experienced the ATs differently, both between countries and between distinct contextual settings. For instance, in Taiwan the utilization of the exergame to train fine motor skills was valued since it improved the skills of PwD, yet there was a need for actual integration in the DSS dashboard. Both in Taiwan and Italy, the exergame WhizToys stimulated physical activity and the PwD thought it was fun to play. However, some technical issues still need to be improved. The senior tablet was also experienced positively both in the Netherlands and Taiwan and could serve to support mediated social contacts. However, most PwD in the Netherlands were not able to interact with the tablet. This was likely due to the (later) stage of dementia they were in. It should be noted that such an AT is specifically targeted at PwD in the early- to mid-phases of dementia (Ipakchian Askari et al., 2024). In the Netherlands and Italy, the deployment of the GPS AT was perceived as instrumental in fostering safe mobility among PwD, thus aligning with broader initiatives aimed at promoting autonomy and mobility within care settings, as elucidated by Buimer et al. (2020) and Sayeh et al. (2022). However, the size and limited portability of the technology posed practical barriers within care practices, prompting suggestions for embedding GPS functionalities within inconspicuous objects to mitigate the risk of misplacement or forgetfulness. However, disguising a GPS as another object raises an ethical dilemma. Depending on the possible health and safety risks, recommending a GPS for a PwD might outweigh the privacy concerns. Therefore, it is of importance to balance the pros and cons and to involve the PwD in the decision process, considering their needs and preferences when designing technology.

Both FCs and ICs valued the use of lifestyle monitoring to support in the early detection of a bladder infection or notification of fall incidents. The 3D fall sensor was used in Italy and was highly valued. A constraint was that the technology could only monitor falls in a limited area. Therefore, multiple technologies were needed to cover a whole house. Finally, the medicine dispenser was valued by all users in the Netherlands, also because it directly supported the PwD instead of just monitoring them. The medicine dispenser could not be used in Italy and Taiwan, since there was no supporting process by pharmacies that could provide medication rolls for the medicine dispenser.

The formative Alpha evaluation study of the DSS and ATs was conducted in three different countries, cultures and care contexts, involving different care professionals and PwD with different GDS cognitive dementia stages (see, Reisberg et al., 1982). The results show that for a DSS to have an added value in the care process, its functionalities and design should be tailored to these different contexts and users. For instance, there should be customization regarding the specific care technologies to be used and data to be collected (see Berridge et al., 2021). Other aspects that should be customized are the data processing by AI, the granting of access to the data and AI-based insights, the way that AI-based insights are explained to the users (see Du et al., 2022), and the extent in which AI-DSSs proactively advise caregivers about care needs and strategies. Carers from all three countries expressed their interest in the DSS and wanted to gain insights into the behavior of PwD for prevention purposes. However, the type of information they were interested in differed. Dutch FCs were mainly interested in monitoring daily behavior such as sleep and eating patterns, while carers from Italy and Taiwan were mainly interested in monitoring the progress that PwDs made in terms of physical activity. The difference was likely related to the intramural care context in Italy and Taiwan and the extramural care context in the Netherlands where PwDs live independently at home. In the Netherlands, the current policy theme in long-term care is “self if possible”, “home if possible”, and “digital if possible” (Rijksoverheid, 2022). Care will be provided increasingly- and mostly at the homes of people, possibly supported by ATs. This transition from intramural care to extramural (home) care was also reflected by the perceptions of FCs from the Netherlands. In particular, they perceived remote monitoring as valuable because the DSS could provide insights that could be collected even when they were not physically present in the house. In the Netherlands, FCs also specifically valued an integration between the DSS and the Electronic Health Record (EHR), as this contextual information is necessary to make useful interpretations of the data shown in the DSS. In the Netherlands, all hospitals and most care organizations use an EHR. An integration between the DSS, the data from and to the ATs and the EHR is in line with the goals of the iterative DSS developments and results presented in this paper. However, interoperability between the DSS and the EHR was not specifically emphasized in the other countries. In Italy this was possibly because of the limited general use of an EHR (HIMSS Italian Community, 2021). Although Taiwan has achieved high adoption of the EHR, it is still a challenge for meaningful use of EHRs among hospitals and clinics (Wen et al., 2019).

Overall, the results illustrate the importance of offering the possibility to customize the DSS depending on the context of use and specific needs of the users within this context. This customization could for example be by varying between functionalities and certain (health) information in the dashboard between an intramural- and extramural care context. As advocated by Cahill et al. (2017), in an intramural care context, technology has a role in terms of supporting the wellbeing of both PwDs and staff alike. This could enable life/job satisfaction and social participation, and foster an environment that provides a sense of purpose for all (i.e., residents, staff and families). The usability of the DSS dashboard differed for each type of caregiver in terms of the details provided by the dashboard and in terms of frequency of use. From the interviews it can be concluded that ICs might not need the level of detail that is currently (meant to be) provided by the dashboard. It is important to determine which data should be shared with the ICs, especially when taking into account who is responsible for monitoring the dashboard. Then there is also a distinction to be made between FCs and case managers dementia. From the qualitative results, it is seen that professional carers—in particular, case managers dementia from the Netherlands—are reluctant to use the DSS dashboard as currently intended. Checking the dashboard regularly and having to act on it on a frequent basis, could be overwhelming and too demanding on top of their existing responsibilities. For this reason, it would make sense to consider developing different versions of the DSS dashboard to fit each caregiver's profile. It would also be beneficial to look at the options to create a separate dashboard for FCs, ICs, and case managers. In contrast, it is important to keep in mind which collaboration is necessary to intervene based on information gathered in the DSS dashboard. There should be a clear division in the responsibilities and the DSS dashboard should support this. For example, by showing notifications to the person who is expected to respond to specific insights. Nevertheless, full customization up to the level of individual users might be at odds with the need to offer somewhat standardized solutions that are universally applicable and foster scalability (Peine and Moors, 2015).

In summary, although the overall experiences were positive, some improvements could be made, thereby optimizing the usability and effectiveness of the DSS system. ICs and FCs did not raise problems related to usability, although they did not use the DSS often and had not explored all the functions. Future Beta developments and a (longitudinal) summative evaluation will provide more insights into the expected outcomes when using the DSS dashboard in context with real-time data from a variety of ATs.

### 4.3 Limitations

No study comes without limitations. The main limitations of the present study were the relatively small sample size, an unbalanced study design, and most importantly, the current development stage of the protype of the DSS. To give participants an impression of the possibilities of the DSS and gather their views at this stage, the DSS used dummy data and working AI algorithms were not yet integrated. The DSS dashboard that was tested had some usability issues. In addition, several ATs were not yet connected to the cloud-based server and the stability of the software also needed to be improved.

In the current study, few explicit questions were asked regarding the broader social and ethical implications of using the DSS. Due to the impact that the use of AI-based technologies may have on people's lives and caregivers' work, such implications need to be assessed and addressed at an early stage of their development. Otherwise, resistance might emerge during implementation. Future studies could examine more systematically and in more depth how different users and other stakeholders perceive and experience both the positive and potentially unintended effects of using DSSs on values such as autonomy, privacy, transparency, and equality. Furthermore, it should be investigated how the ways in which these DSSs support the care process and interact with users can be flexibly refined “in context” (Lukkien et al., 2023). Moreover, within long-term care, an increasing number of caregiving responsibilities might transition to informal care networks. When ICs have access to a DSS, such as the one used in this study, they get more knowledge of the person they are caring for. This knowledge could help them to co-create the care together with formal caregivers. The DSS could serve as a conversational guide during this co-creation process.

Anticipated progress in AI suggests a growing role of DSSs in proactively supporting caregivers and PwD in shared decision-making about person-centered care strategies by harnessing relevant data through machine learning. In this sense, data collected from various ATs which is made available centrally in a dashboard, can be increasingly utilized to, e.g., perform predictive analyses on risk factors or to perform prescriptive analyses regarding the mitigation of risks and person-centered care strategies (El Morr and Ali-Hassan, 2019; Mosavi and Santos, 2020). Moreover, it is important that in the development of a DSS, the data is validated in order to ensure that the DSS can provide accurate and reliable data.

### 4.4 Future research

As discussed before, further development of the DSS entails integration of actual data from the ATs and running further evaluations aimed at refining and optimizing the design(s). After the Alpha evaluation, about all participants were willing to continue using the HAAL technologies and dashboard and were enrolled for the follow-up summative evaluation (Beta evaluation) of the HAAL project. For future research it would be beneficial to extend the evaluation period with a larger sample size, as this could enhance the generalizability of the findings and provide more in-depth insight into the long-term effectiveness and sustainability of the use of the DSS. Additionally, in such a follow-up study it is also needed to gain more insights into the perceptions, opinions, beliefs and attitudes of PwD regarding a DDS designed to monitor their behavioral patterns and predict their health situation. Questions can be raised about what is needed to ensure a good balance between values as privacy, autonomy, and care and support needs by ICs and FCs for monitoring and health prevention. Furthermore, ICs and PwD are prospective end-users of the DSS, in particular with increasing self-care and home-care practices. This entails further developments and design refinements of the DSS with genuine iterative involvement of these intended user groups. It is also relevant to study which ATs and which data PwD and their carers prefer to get access to in a DSS. Moreover, it would be beneficial to explore opportunities to integrate the DSS and AT bundle with telehealth services to enable remote monitoring, consultation, and intervention delivery. This could enhance access to care for PwD living in remote or underserved areas and improve care coordination among healthcare providers. Finally, future studies should also focus on cost-effectiveness. Cost-benefit analyses are highly needed in a time with increasing health care costs and a decreasing workforce of FCs.

## 5 Conclusion

In general, carers and PwD expressed a positive and hopeful attitude toward the DSS dashboard and AT bundle. Despite the fact that the dashboard currently represents an initial Alpha prototype, its potential is seen in efficiently presenting the overall wellbeing of PwD to both FCs and ICs. This, in turn, holds promise for augmenting caregivers' understanding of people's care needs, ultimately benefiting their journey. In the paper we reflected on the formative evaluation results on the usability and acceptability of the prototype by carers and PwD. The insights are used to further improve the dashboard. Furthermore, the findings contribute to the existing body of knowledge surrounding DSS deployment within care contexts, shedding light on the potential impact of DSS utilization on acceptability and perceived workload among both FCs and ICs. The insights garnered from this study can support the effective and responsible development of such DSS solutions, ultimately reducing the workload of carers and supporting the autonomy and QoL of PwD.

## Data availability statement

The raw data supporting the conclusions of this article will be made available by the authors, without undue reservation.

## Ethics statement

The study was approved by the Ethic Committees in the individual countries and the pilot sites (Netherlands, NW2023-13; Italy, INRCA n. 3750/2023; NCKU B-ER-112-026). The principles of the Declaration of Helsinki and Good Clinical Practice guidelines were adhered to. Personal data collected during the trial was handled and stored in accordance with the General Data Protection Regulation (GDPR) 2018 (Protection, 2018). The studies were conducted in accordance with the local legislation and institutional requirements. Written informed consent for participation in this study was provided by the participants' legal guardians/next of kin. Written informed consent was obtained from the individual(s) for the publication of any potentially identifiable images or data included in this article.

## Author contributions

HN: Conceptualization, Formal analysis, Funding acquisition, Investigation, Methodology, Project administration, Resources, Supervision, Validation, Visualization, Writing – original draft, Writing – review & editing. NS: Formal analysis, Investigation, Supervision, Validation, Writing – original draft. SI: Data curation, Formal analysis, Investigation, Methodology, Supervision, Visualization, Writing – original draft, Writing – review & editing. DL: Data curation, Formal analysis, Investigation, Supervision, Writing – original draft, Writing – review & editing. BH: Formal analysis, Investigation, Supervision, Writing – original draft, Writing – review & editing. NM: Methodology, Resources, Software, Validation, Writing – original draft. SC: Data curation, Software, Supervision, Writing – original draft. GA: Methodology, Validation, Writing – original draft. RB: Methodology, Writing – original draft. AM: Methodology, Writing – original draft. FB: Investigation, Methodology, Writing – original draft. C-JL: Methodology, Validation, Writing – original draft. H-FC: Methodology, Supervision, Validation, Writing – original draft. F-CS: Supervision, Writing – original draft. GR: Writing – review & editing. ET: Writing – review & editing. DB: Investigation, Validation, Writing – original draft. CW: Investigation, Validation, Writing – original draft. Y-LH: Writing – review & editing.

## Footnotes

^1^ https://www.aal-europe.eu/
